# Supplementary material for: Single-cell RNA sequencing reveals the cellular and molecular heterogeneity of treatment-naïve primary osteosarcoma in dogs
Source: Commun Biol. 2024 Apr 24;7:496. doi: 10.1038/s42003-024-06182-w (PMC11043452; doi:10.1038/s42003-024-06182-w)
Supplement: Supplementary file 2 — Supplementary Information [file 42003_2024_6182_MOESM2_ESM.pdf]

Supplemental Figures and Tables for, “Single-cell RNA sequencing reveals the cellular and molecular heterogeneity of treatment-naïve primary osteosarcoma in dogs”

Running title: Canine osteosarcoma scRNA-seq reference

Dylan T. Ammons[a*], Leone S. Hopkins[b], Kathryn E. Cronise[a], Jade Kurihara[b], Daniel P. Regan[a,b], and Steven Dow[a,b*]

[a]Department of Microbiology, Immunology and Pathology, College of Veterinary Medicine and Biomedical Sciences, Colorado State University, Fort Collins, CO

[b]Flint Animal Cancer Center, Department of Clinical Sciences, College of Veterinary Medicine and Biomedical Sciences, Colorado State University, Fort Collins, CO

*Corresponding authors:

Dylan T. Ammons, Department of Microbiology, Immunology and Pathology, College of Veterinary Medicine and Biomedical Sciences, Colorado State University, Fort Collins, CO 80523; e-mail: dylan.ammons@colostate.edu

Steven Dow, Flint Animal Cancer Center, Department of Clinical Sciences, College of Veterinary Medicine and Biomedical Sciences, Colorado State University, Fort Collins, CO 80523; e-mail: steven.dow@colostate.edu

Keywords: single-cell RNA sequencing, canine, osteosarcoma, tumor microenvironment

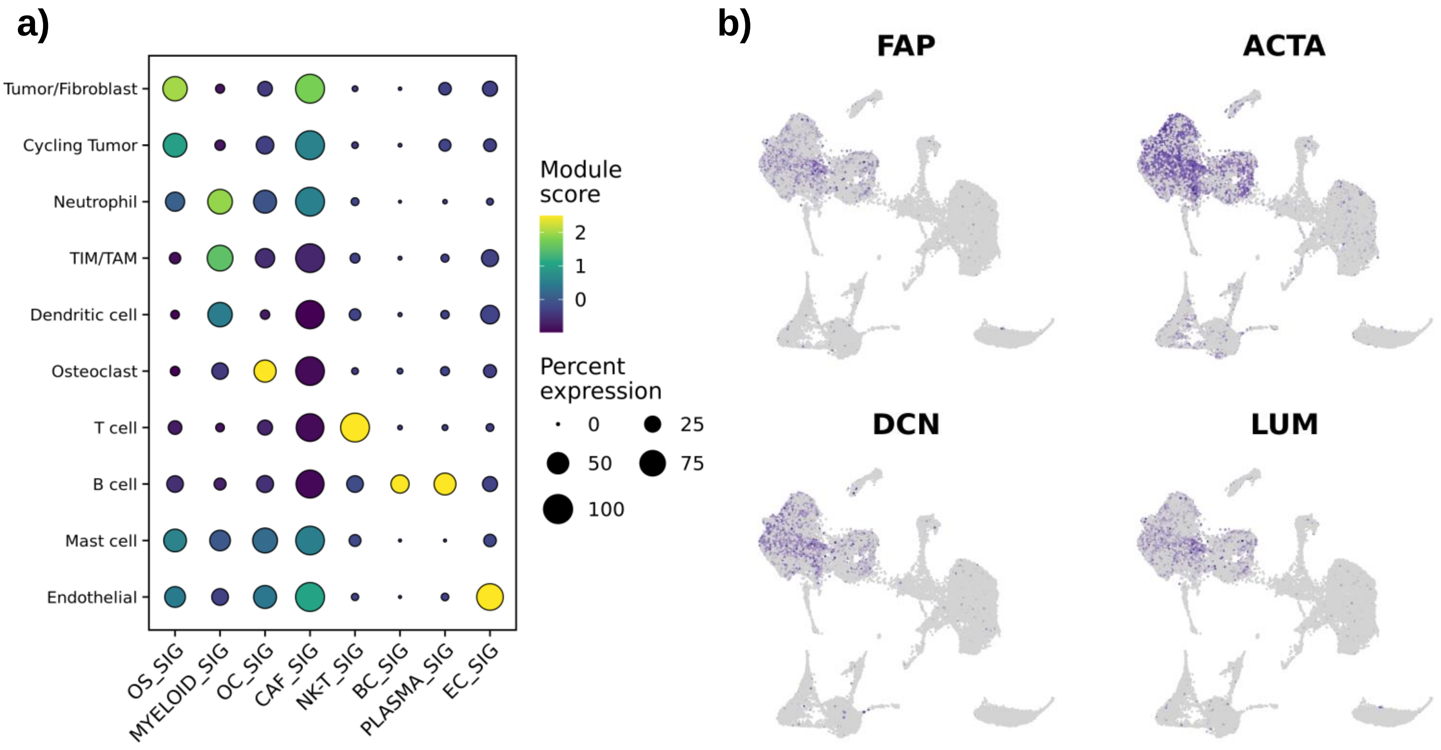

Supplemental figure 1. (**a**) Dot plot depicting the scaled module score of gene lists used to identify cell populations in human osteosarcoma datasets. (**b**) Feature plots depicting the log normalized counts of fibroblast related genes.

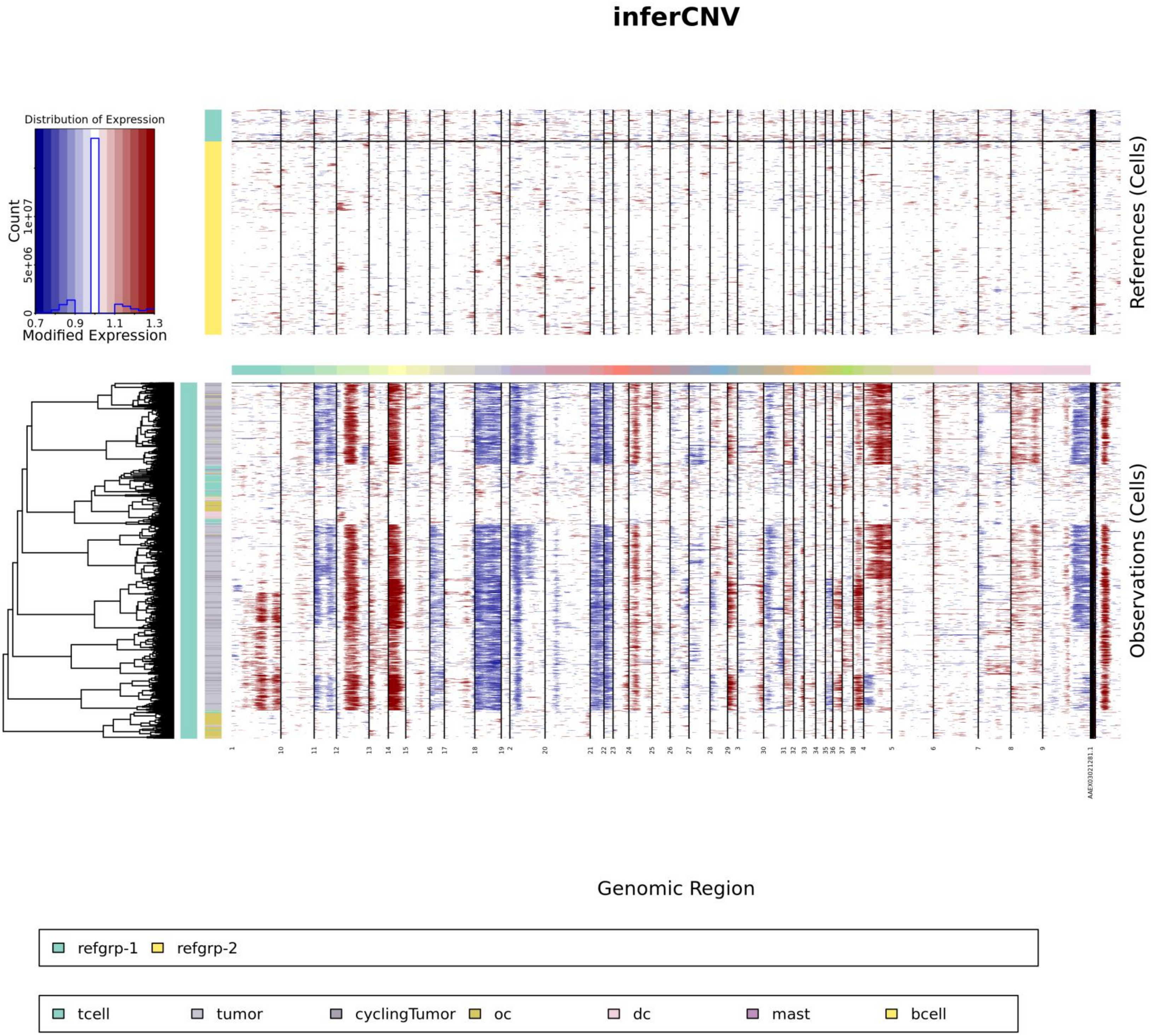

Supplemental figure 2. Heatmap depicting the results of inferCNV for sample Naïve 1_1. Abbreviations: refgrp-1 = Endothelial cells, refgrp-2 = TAMs/TIMs, oc = osteoclast, dc = dendritic cell.

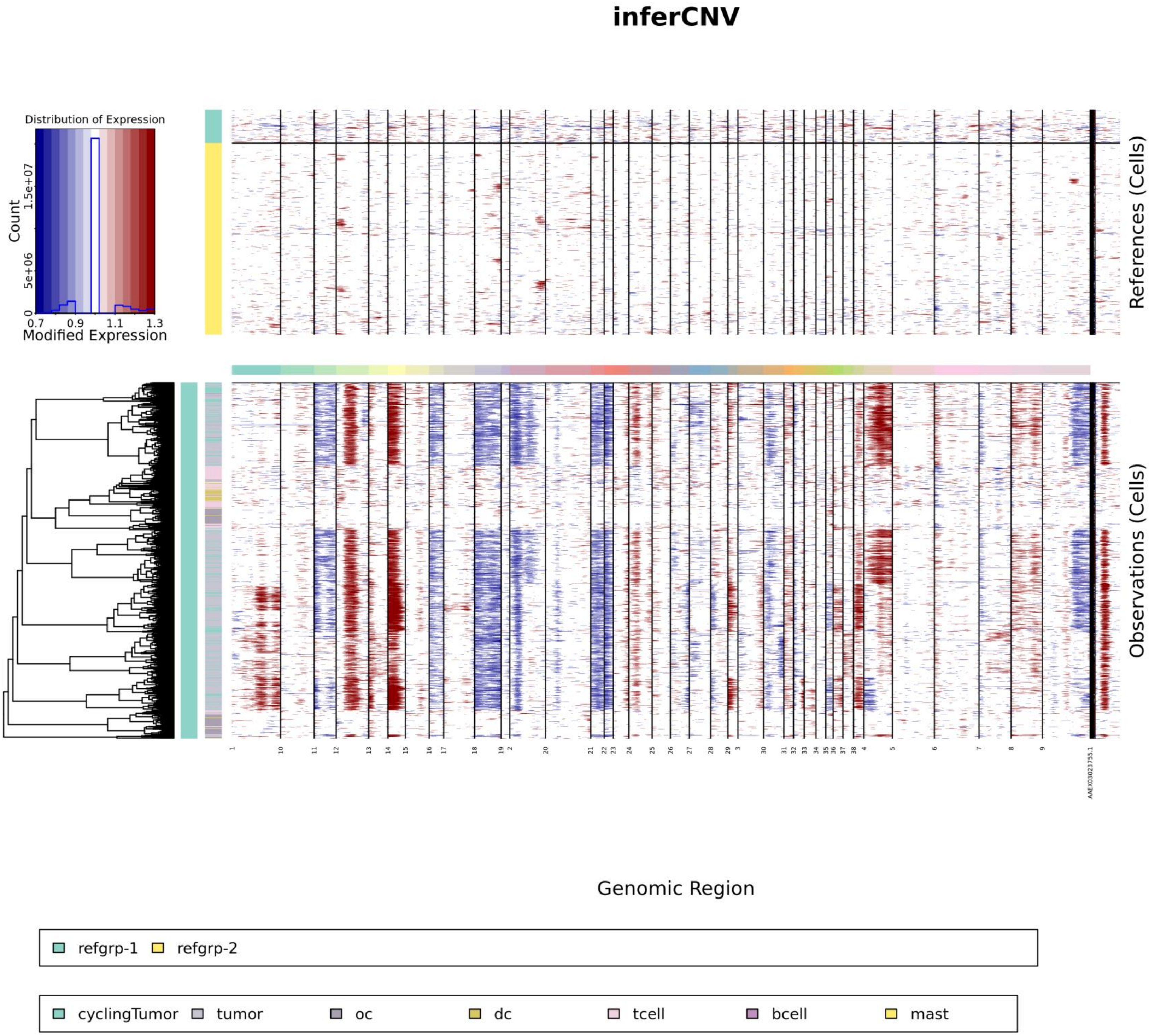

Supplemental figure 3. Heatmap depicting the results of inferCNV for sample Naïve 1_2. Abbreviations: refgrp-1 = Endothelial cells, refgrp-2 = TAMs/TIMs, oc = osteoclast, dc = dendritic cell.

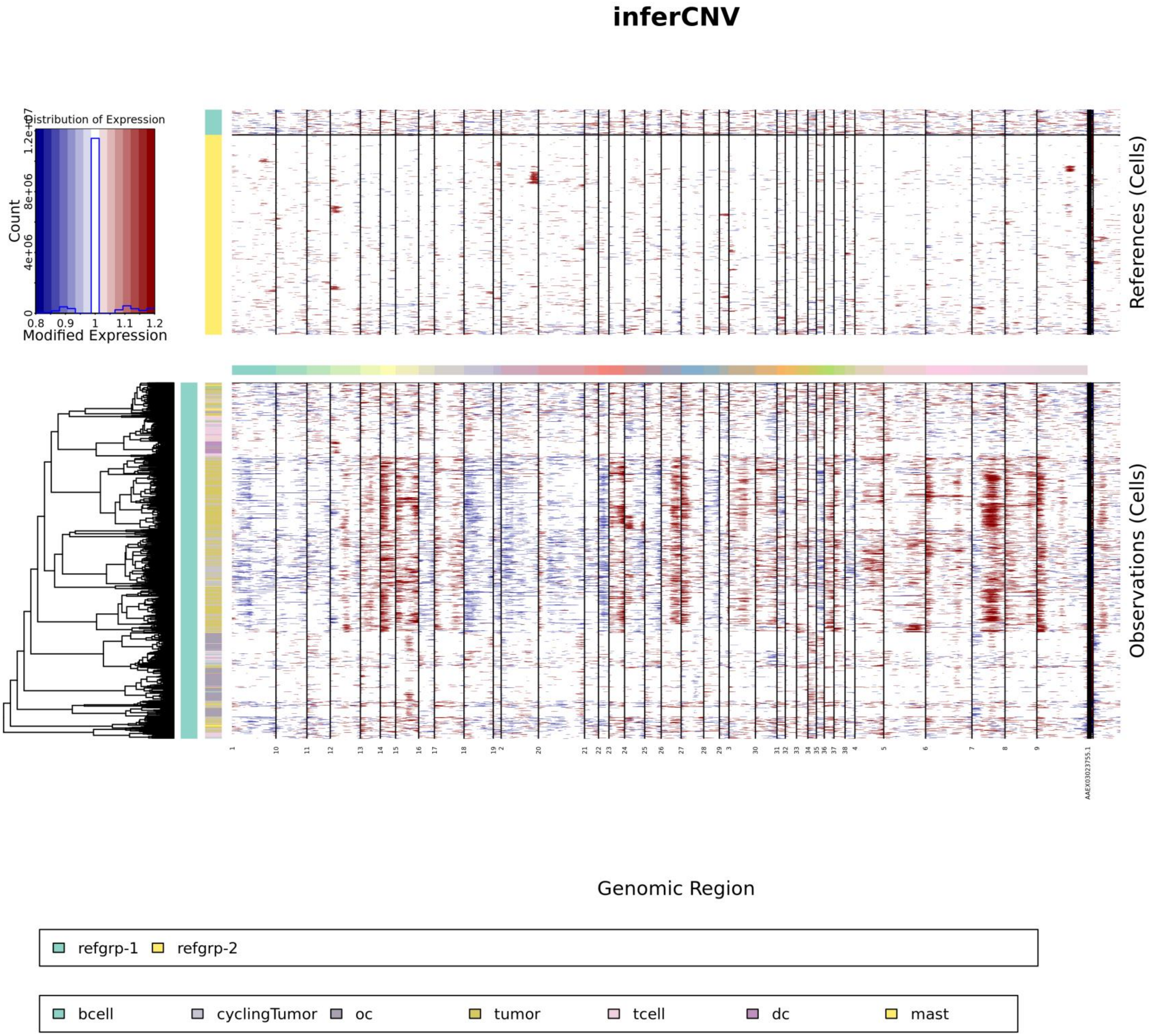

Supplemental figure 4. Heatmap depicting the results of inferCNV for sample Naïve 2_1. Abbreviations: refgrp-1 = Endothelial cells, refgrp-2 = TAMs/TIMs, oc = osteoclast, dc = dendritic cell.

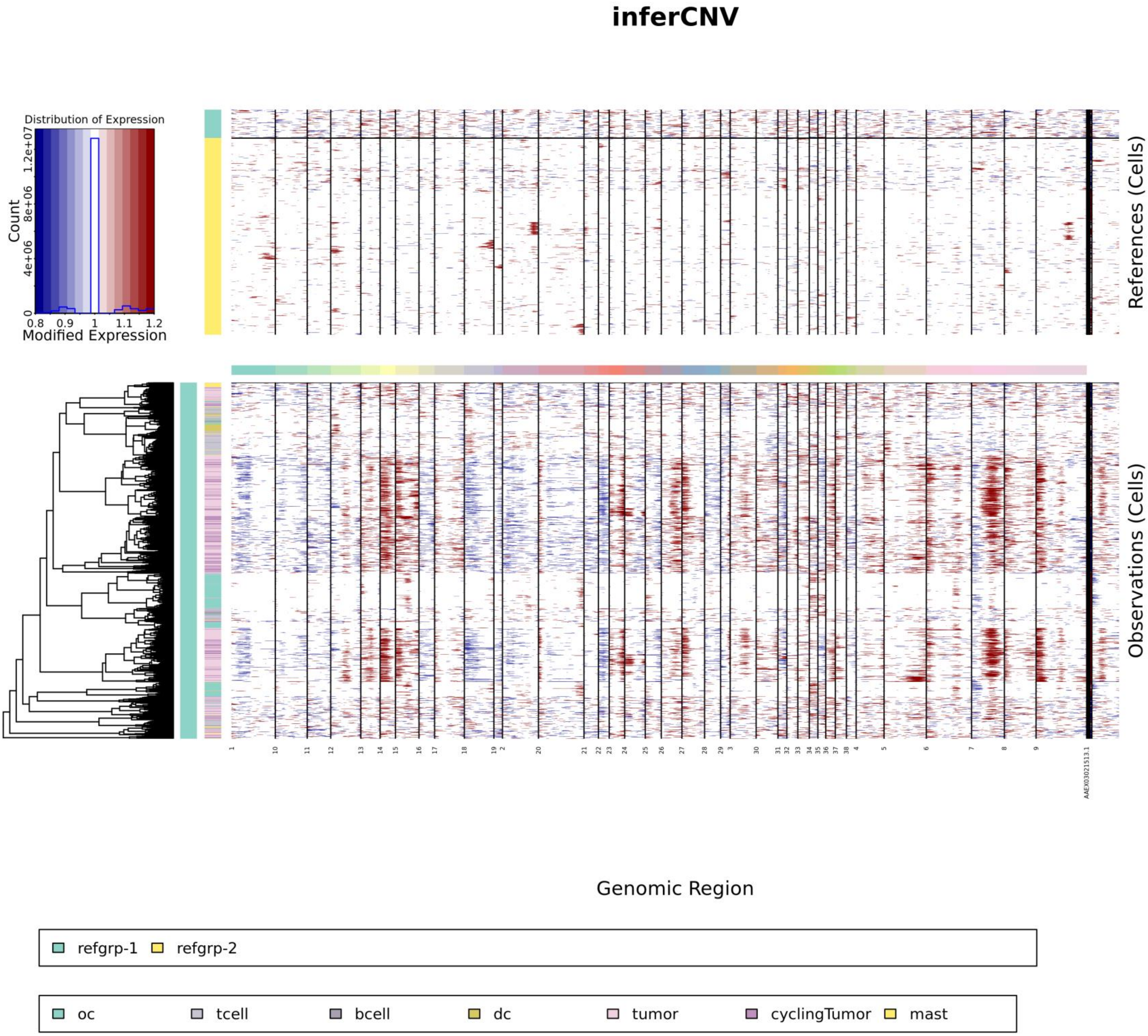

Supplemental figure 5. Heatmap depicting the results of inferCNV for sample Naïve 2_2. Abbreviations: refgrp-1 = Endothelial cells, refgrp-2 = TAMs/TIMs, oc = osteoclast, dc = dendritic cell.

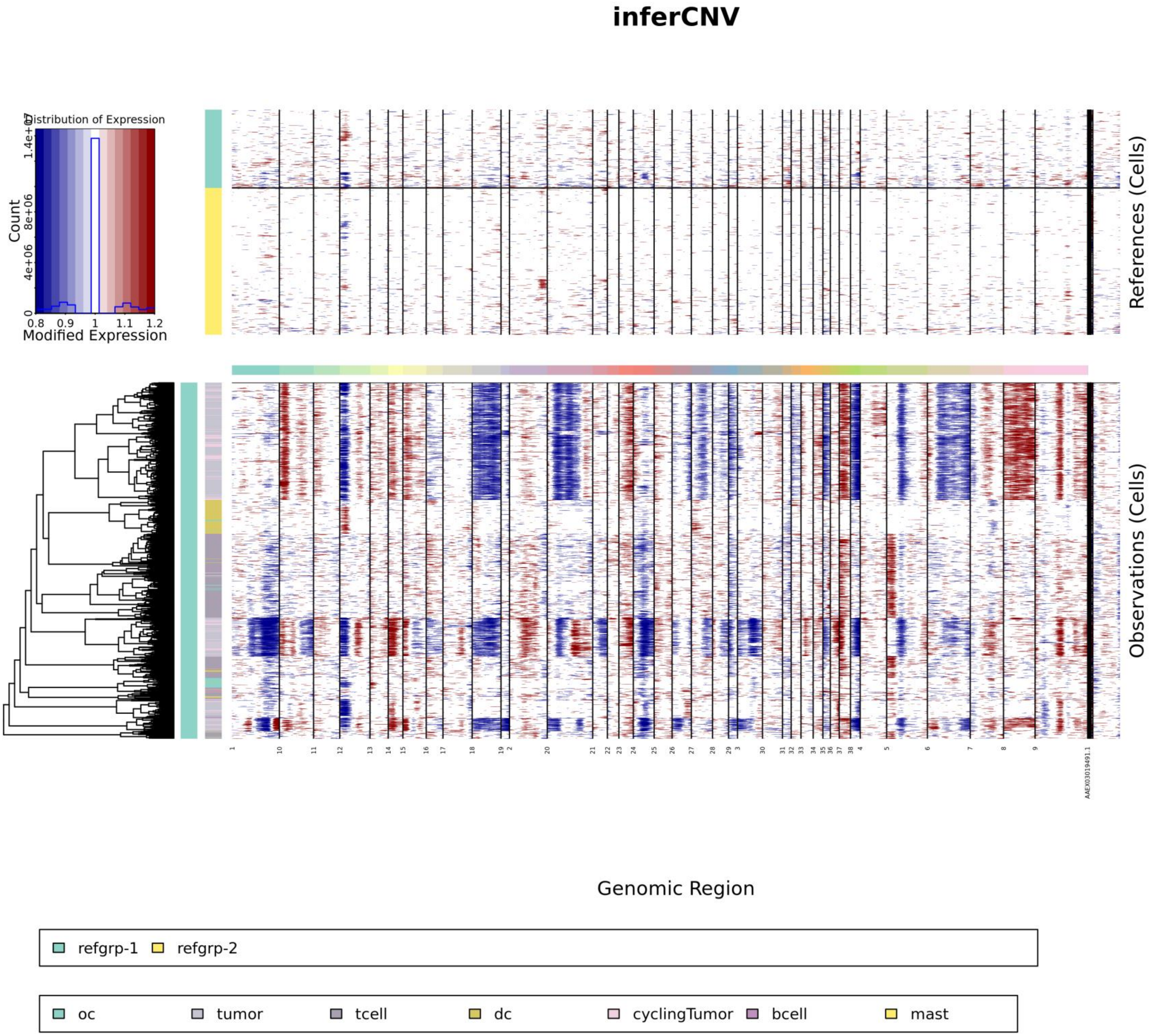

Supplemental figure 6. Heatmap depicting the results of inferCNV for sample Naïve 3. Abbreviations: refgrp-1 = Endothelial cells, refgrp-2 = TAMs/TIMs, oc = osteoclast, dc = dendritic cell.

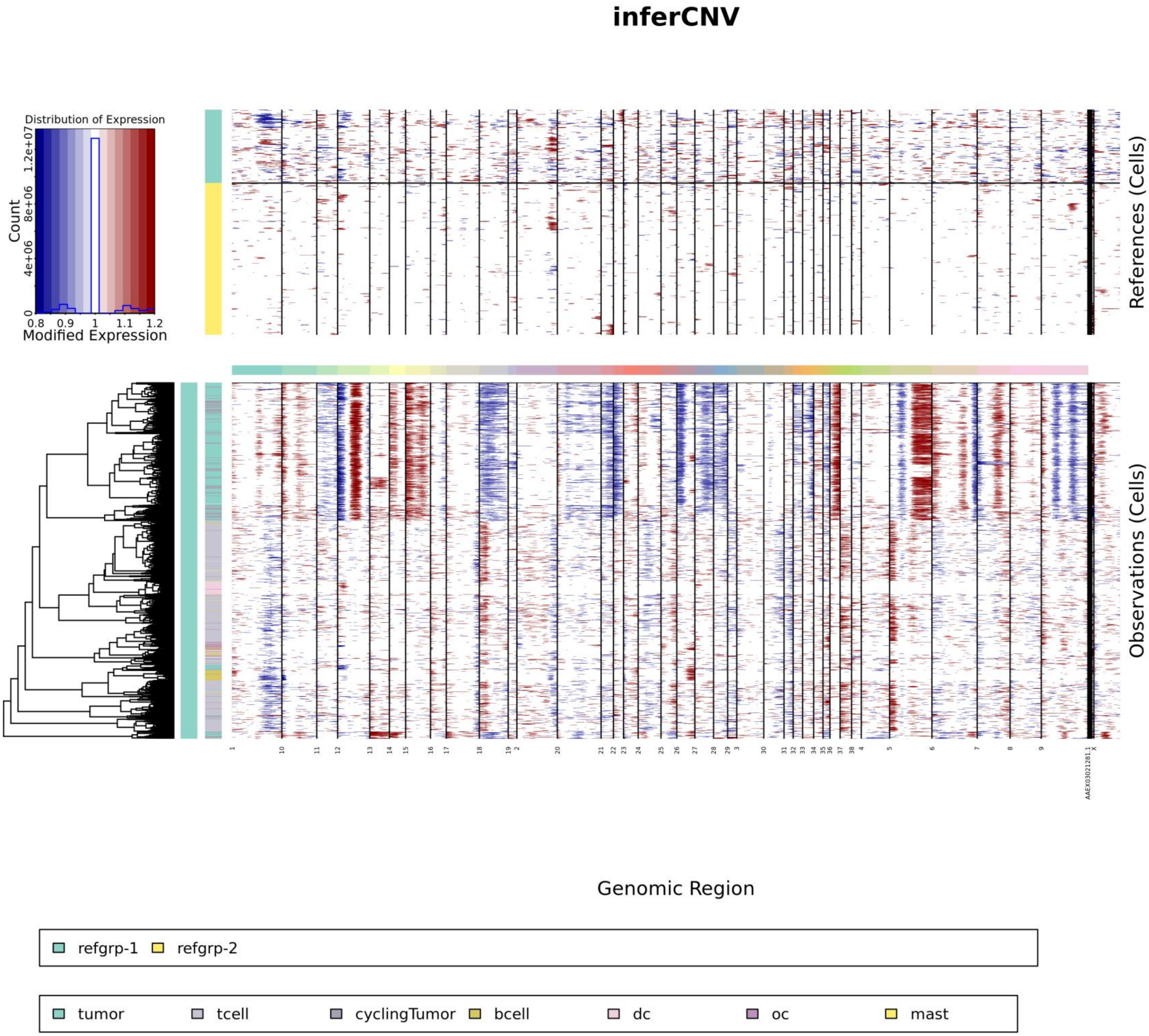

Supplemental figure 7. Heatmap depicting the results of inferCNV for sample Naïve 4. Abbreviations: refgrp-1 = Endothelial cells, refgrp-2 = TAMs/TIMs, oc = osteoclast, dc = dendritic cell.

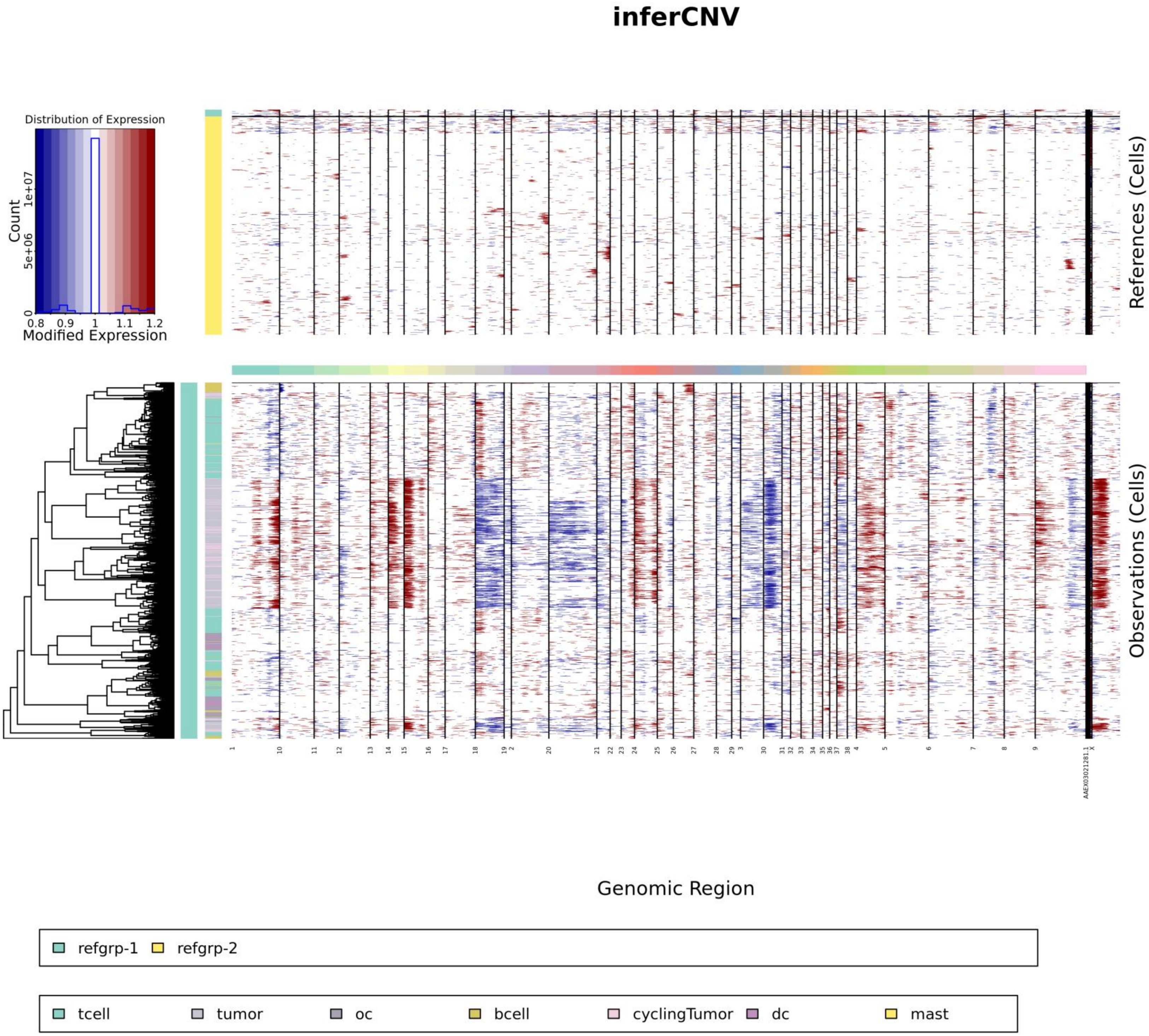

Supplemental figure 8. Heatmap depicting the results of inferCNV for sample Naïve 5. Abbreviations: refgrp-1 = Endothelial cells, refgrp-2 = TAMs/TIMs, oc = osteoclast, dc = dendritic cell.

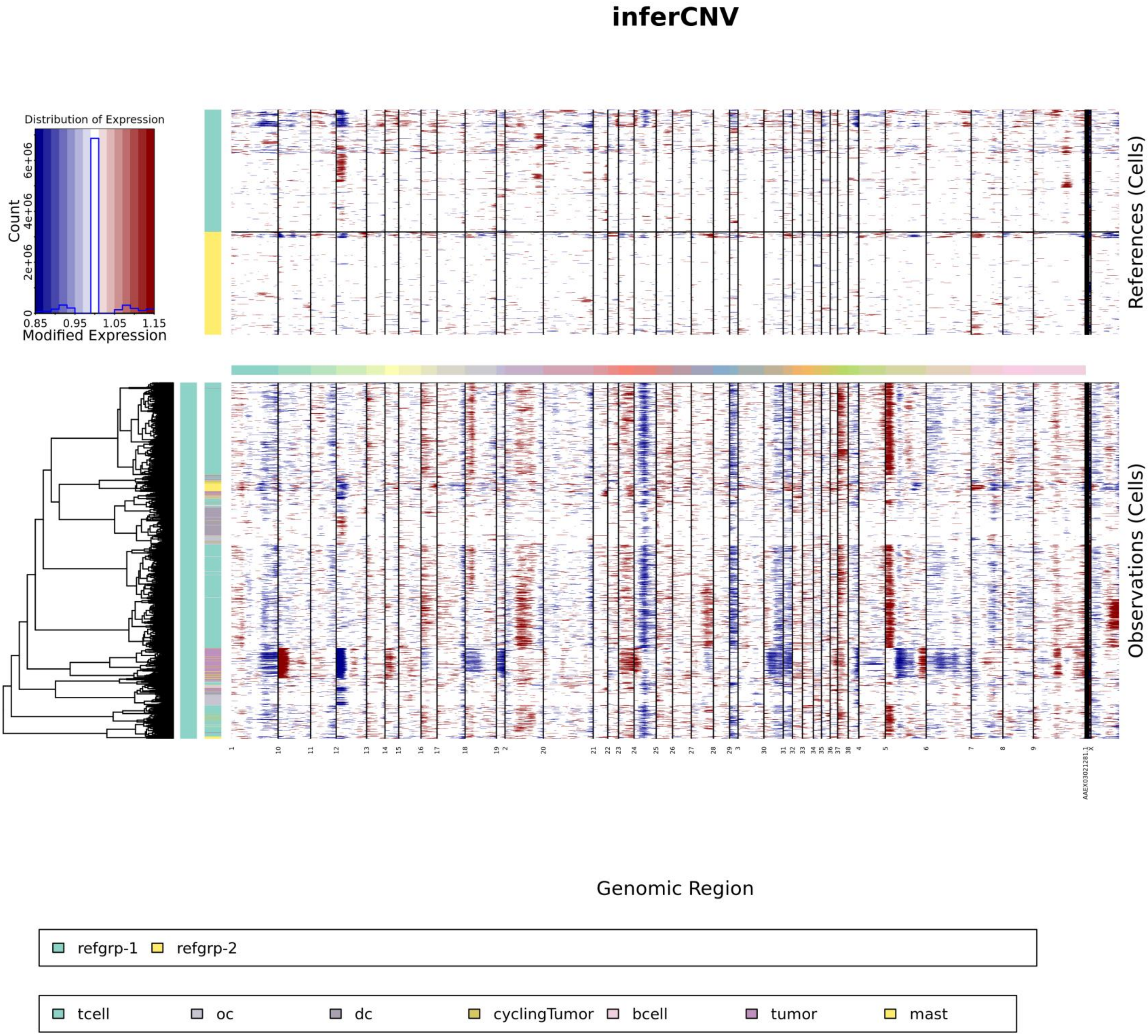

Supplemental figure 9. Heatmap depicting the results of inferCNV for sample Naïve 6. Abbreviations: refgrp-1 = Endothelial cells, refgrp-2 = TAMs/TIMs, oc = osteoclast, dc = dendritic cell.

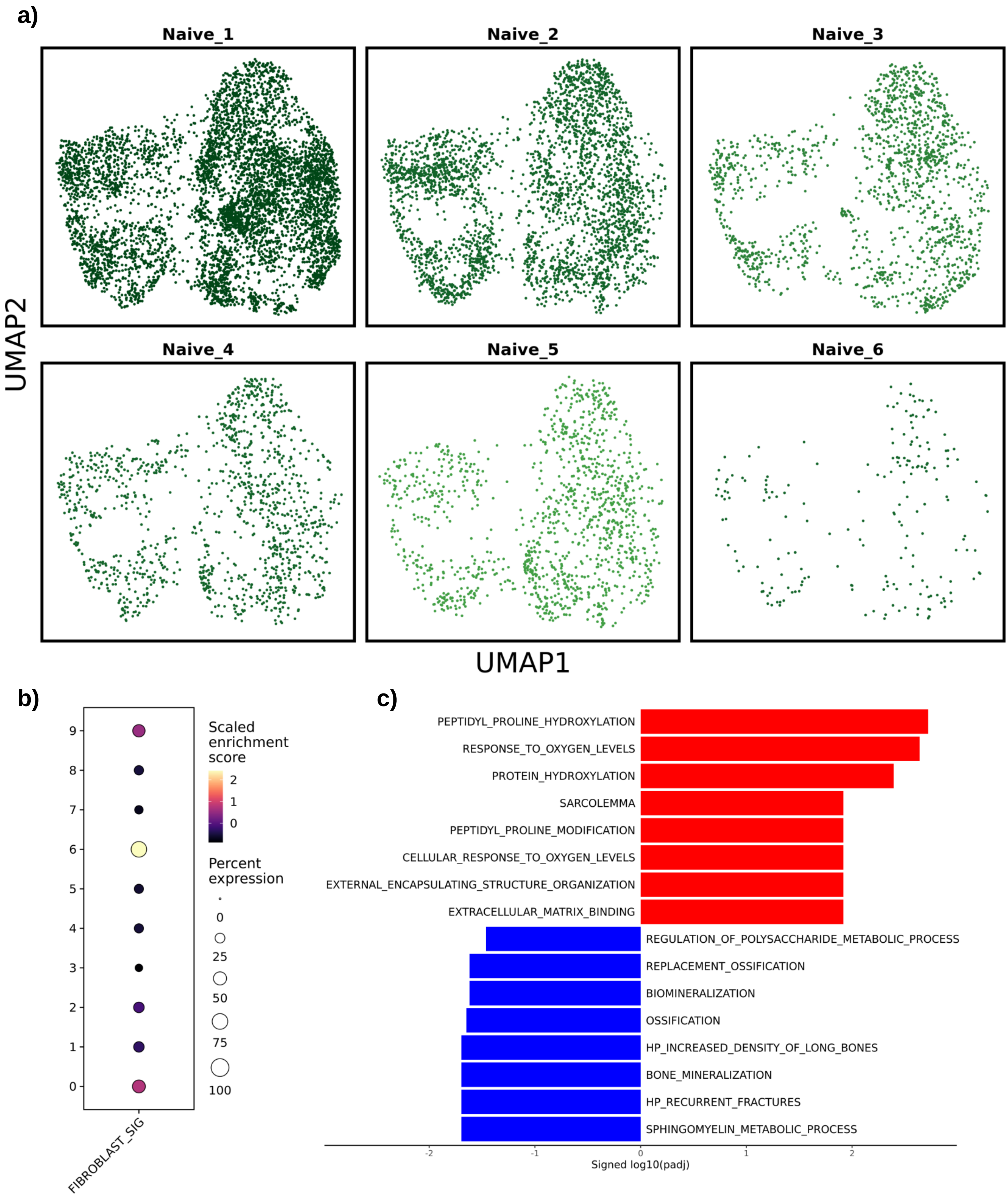

Supplemental figure 10. (**a**) UMAP faceted by dog and downsampled to depict equal numbers of cells from each dog. (**b**) Dot plot depicting the scaled module score of a fibroblast gene list. (**c**) Bar chart depicting the results of gene set enrichment analysis from the differential gene expression analysis of hypoxic osteoblasts (c4) versus non-hypoxic osteoblasts (c0, c1, c2) in Figure 2e.

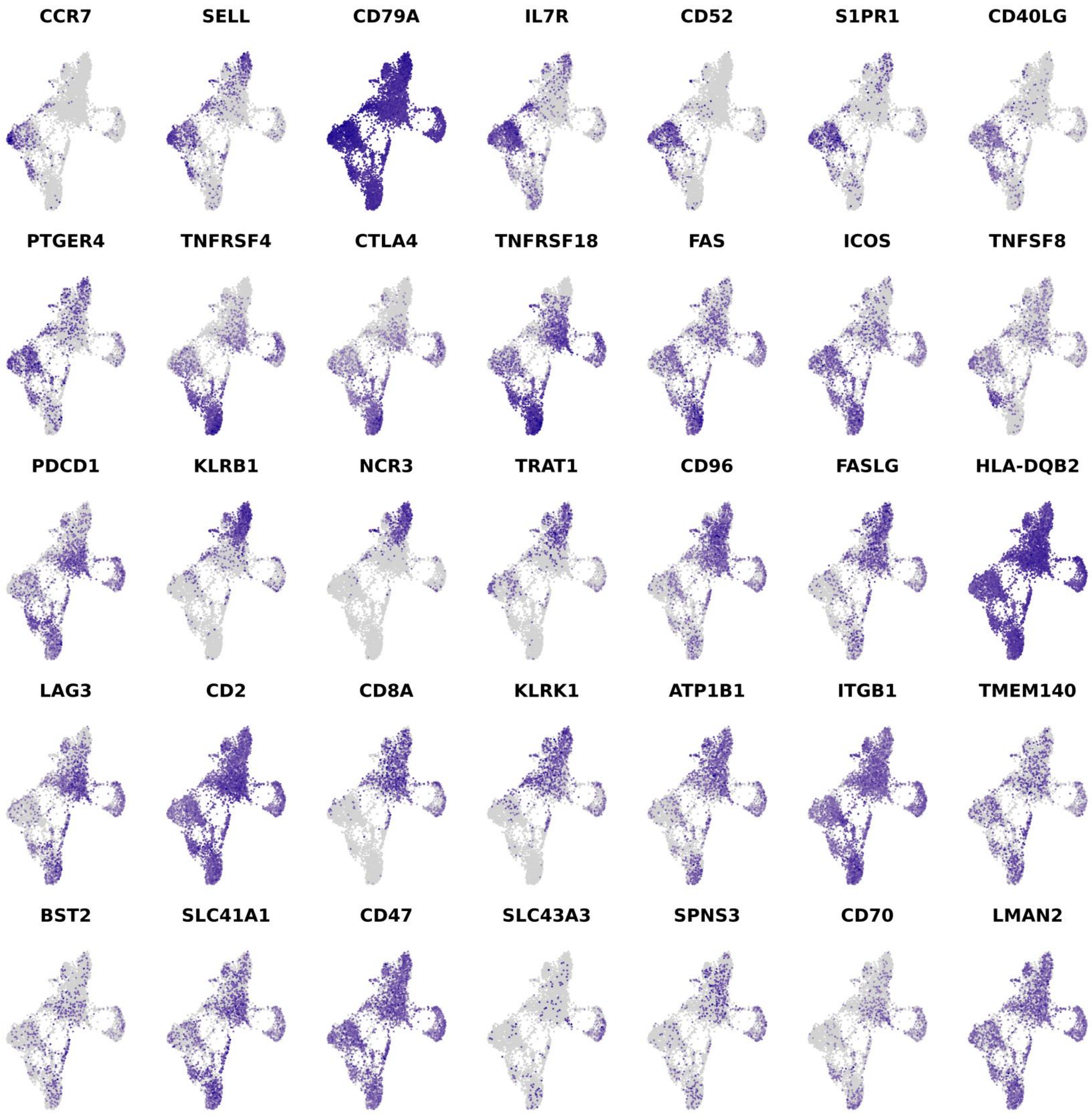

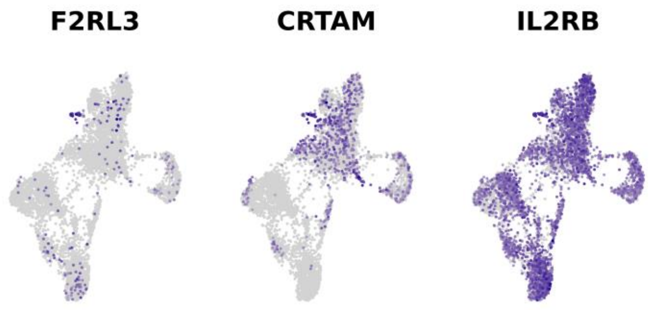

Supplemental figure 11. Feature plots depicting the log normalized counts of features in T cells presented in the surface marker screen heatmap in Figure 3g.

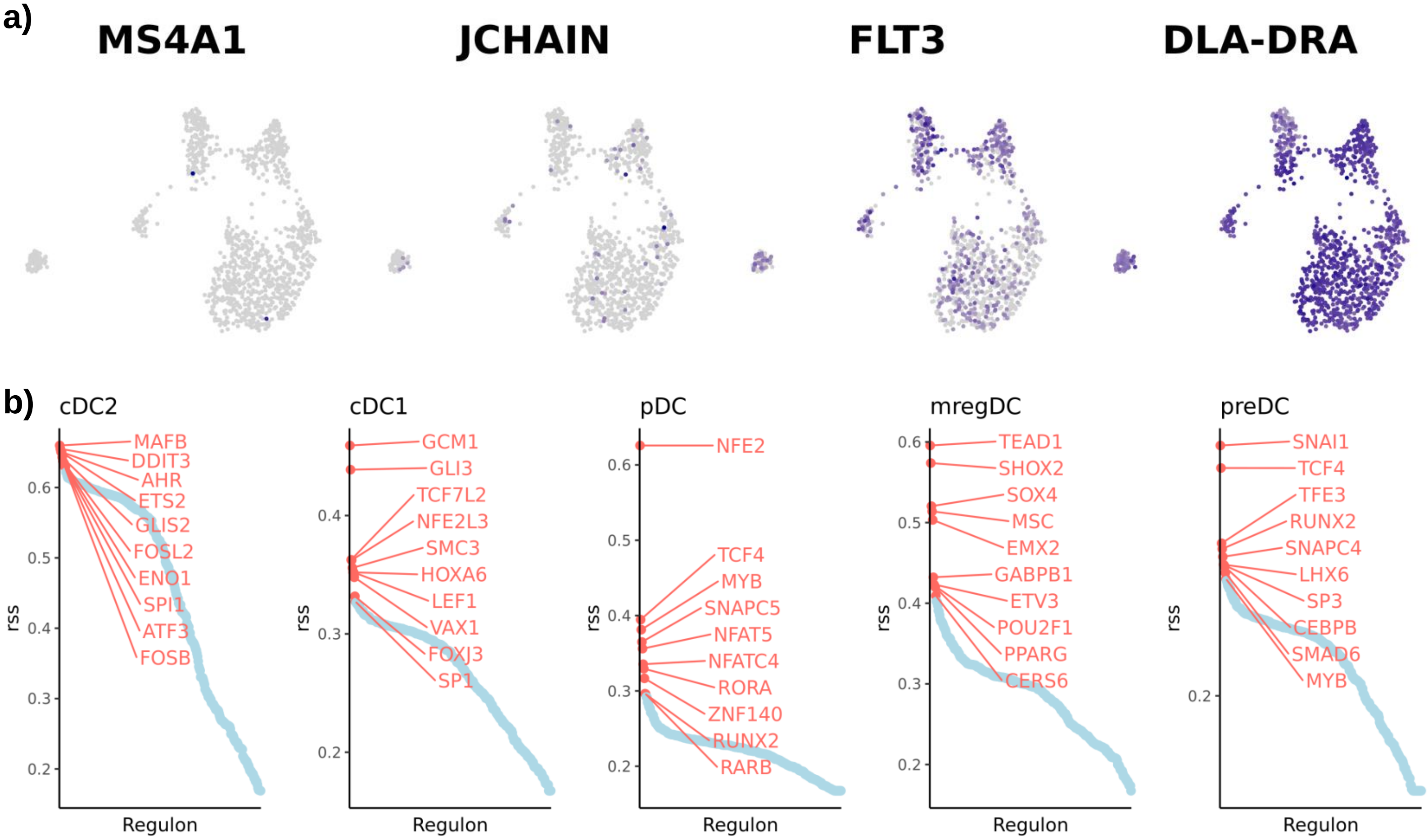

Supplemental figure 12. (**a**) Feature plots depicting the log normalized counts of B cell and dendritic cell associated features. (**b**) Scatter plots depicting regulon specificity score (rss) for active transcription factors in plasmacytoid dendritic cells (pDC), precursor dendritic cells (preDC), conventional DC2s (cDC2s), conventional DC1s (cDC1s), and mature regulatory DCs (mregDCs) as determined using pySCENIC.

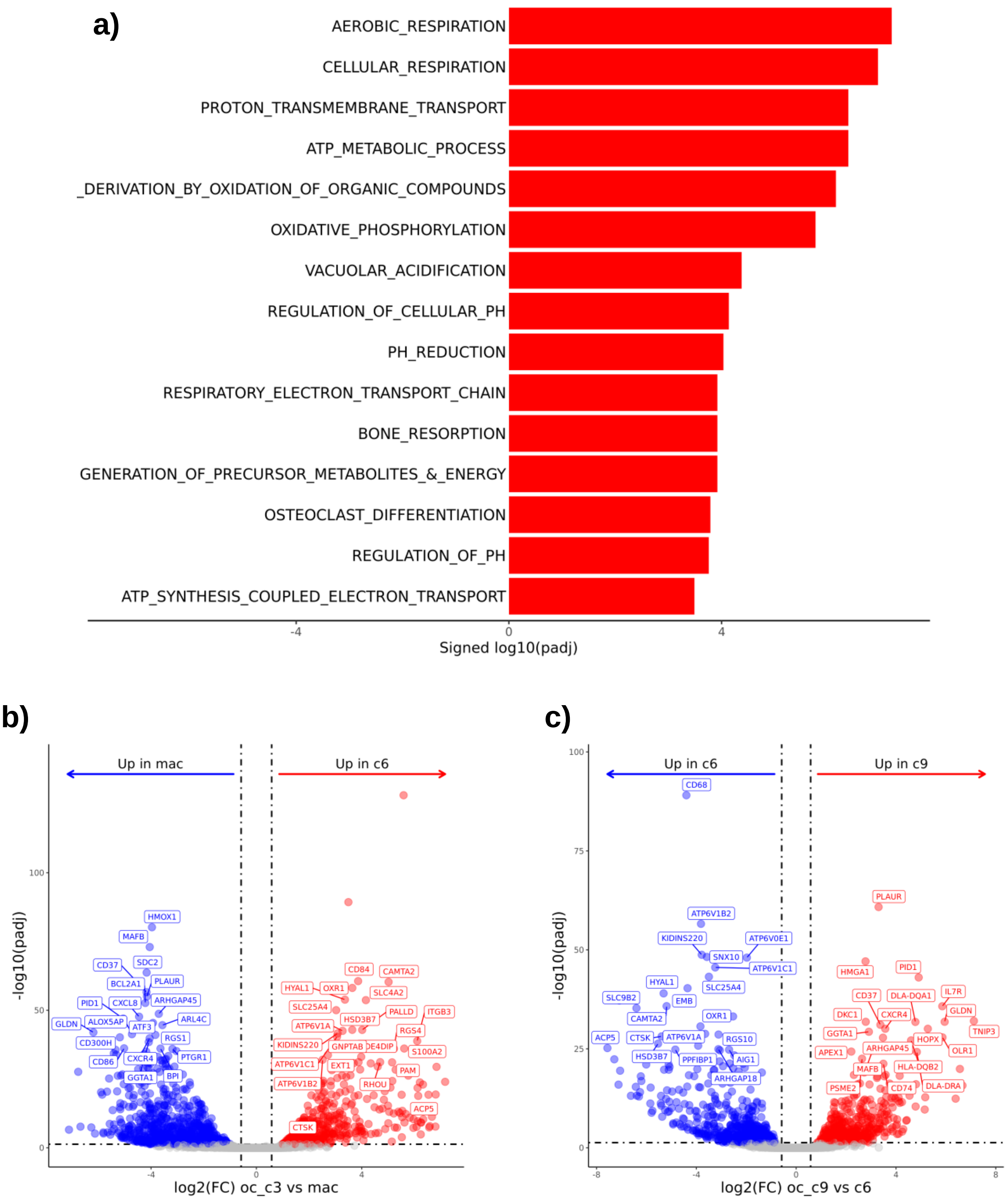

Supplemental figure 13. (**a**) Bar chart depicting the results of gene set enrichment analysis from the differential gene expression analysis Mature OC (c6) versus macrophage (c0, c1, c2, and c3) in Figure 6d. Volcano plot depicting the results of differential gene expression analysis between (**b**) CD320+ OCs versus macrophages (c0, c1, c2, and c3) and (**c**) CD320+ OCs versus Mature OCs.

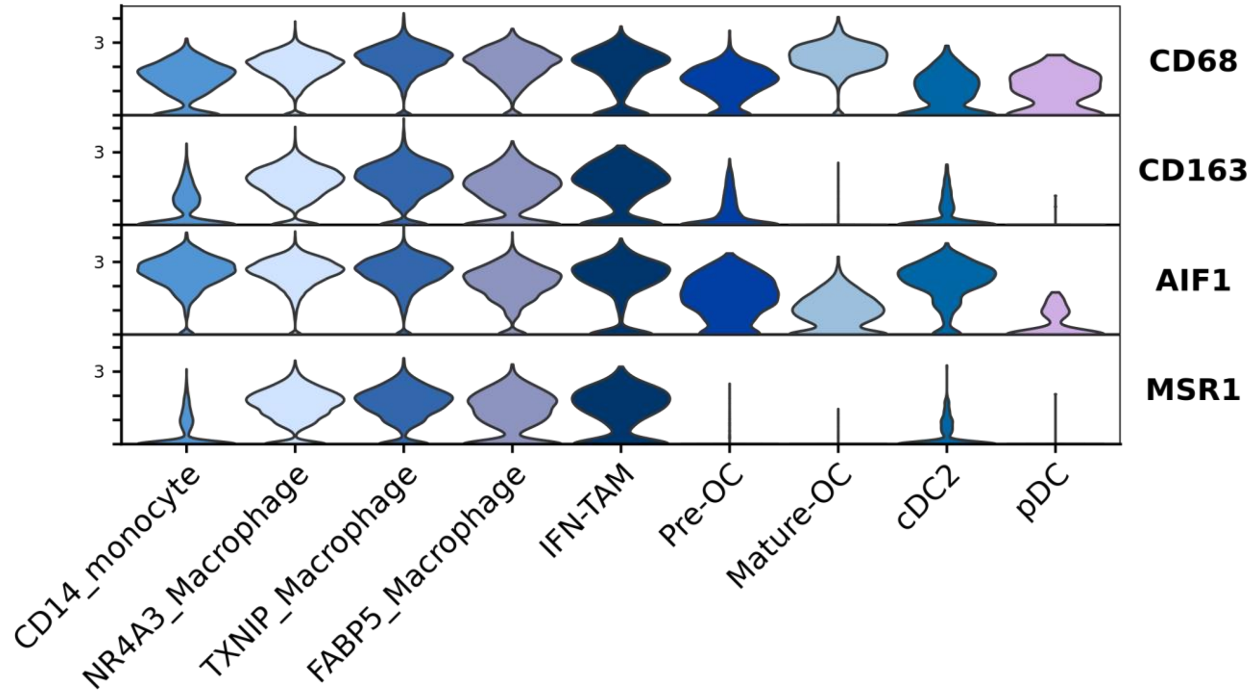

Supplemental figure 14. Violin plots of four immunohistochemistry (IHC) macrophage markers widely used to evaluate macrophage infiltrates in canine and human osteosarcoma. The human data is presented here.

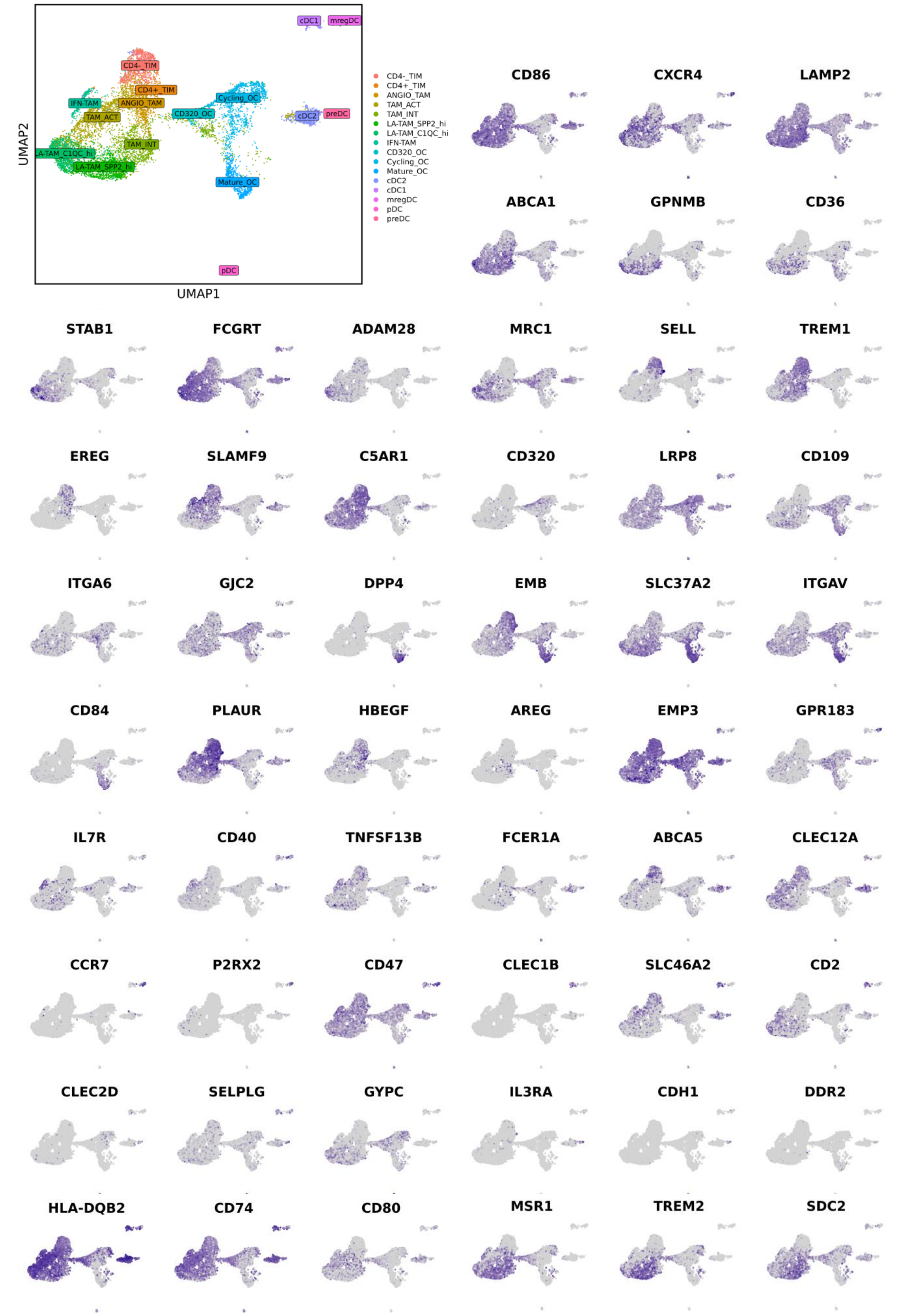

Supplemental figure 15. Feature plots depicting the log normalized counts of features in myeloid cells presented in the surface marker screen heatmap in Figure 7b.

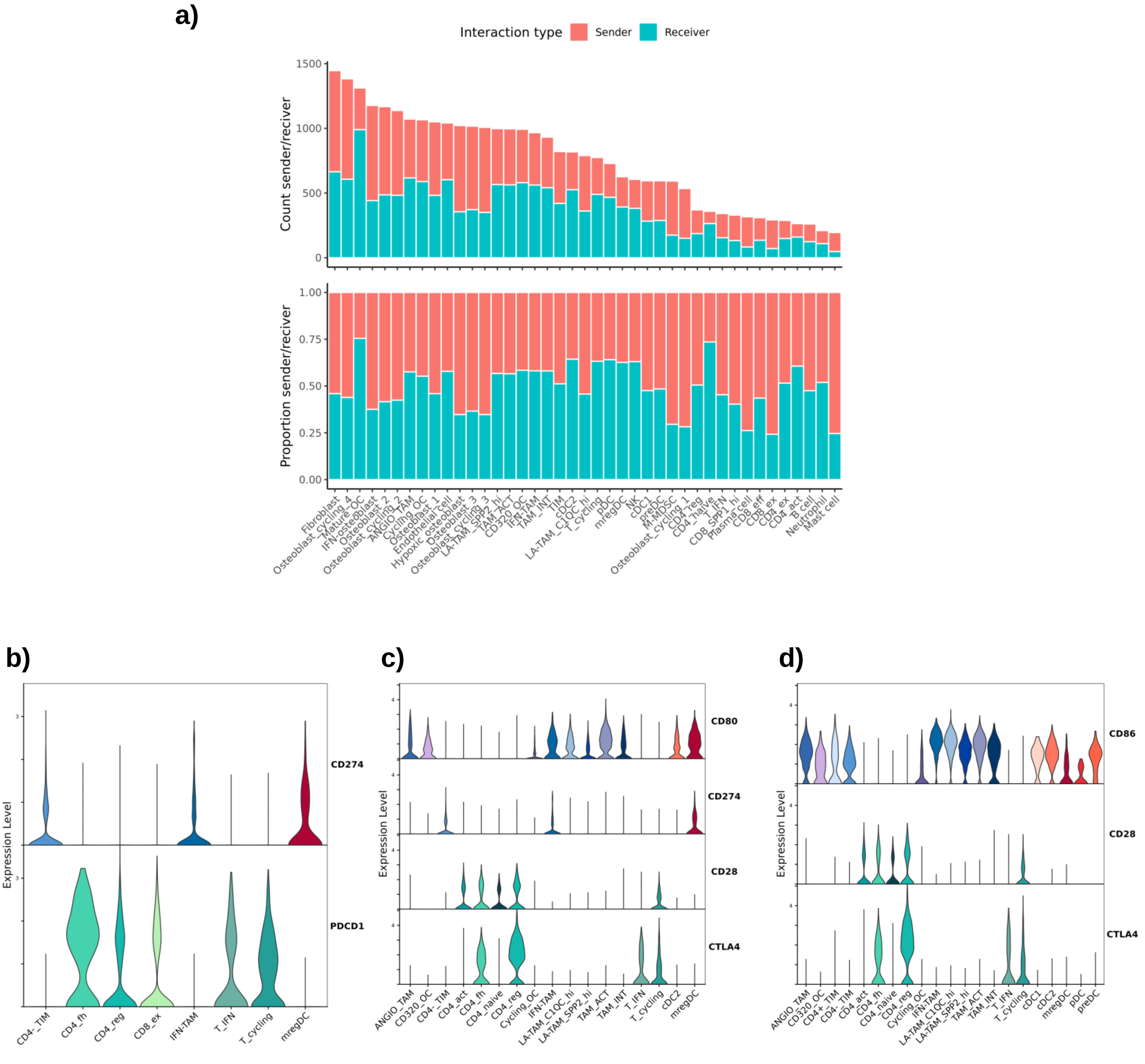

Supplemental figure 16. (**a**) Bar chart depicting the number of CellChat inferred interactions (top) and the percentage interactions in which a cell type is predicted to be sending or receiving an interaction (bottom). Violin plots of features included in the CellChat (**b**) PDL1 network, (**c**) CD80 network, and (**d**) CD86 network.

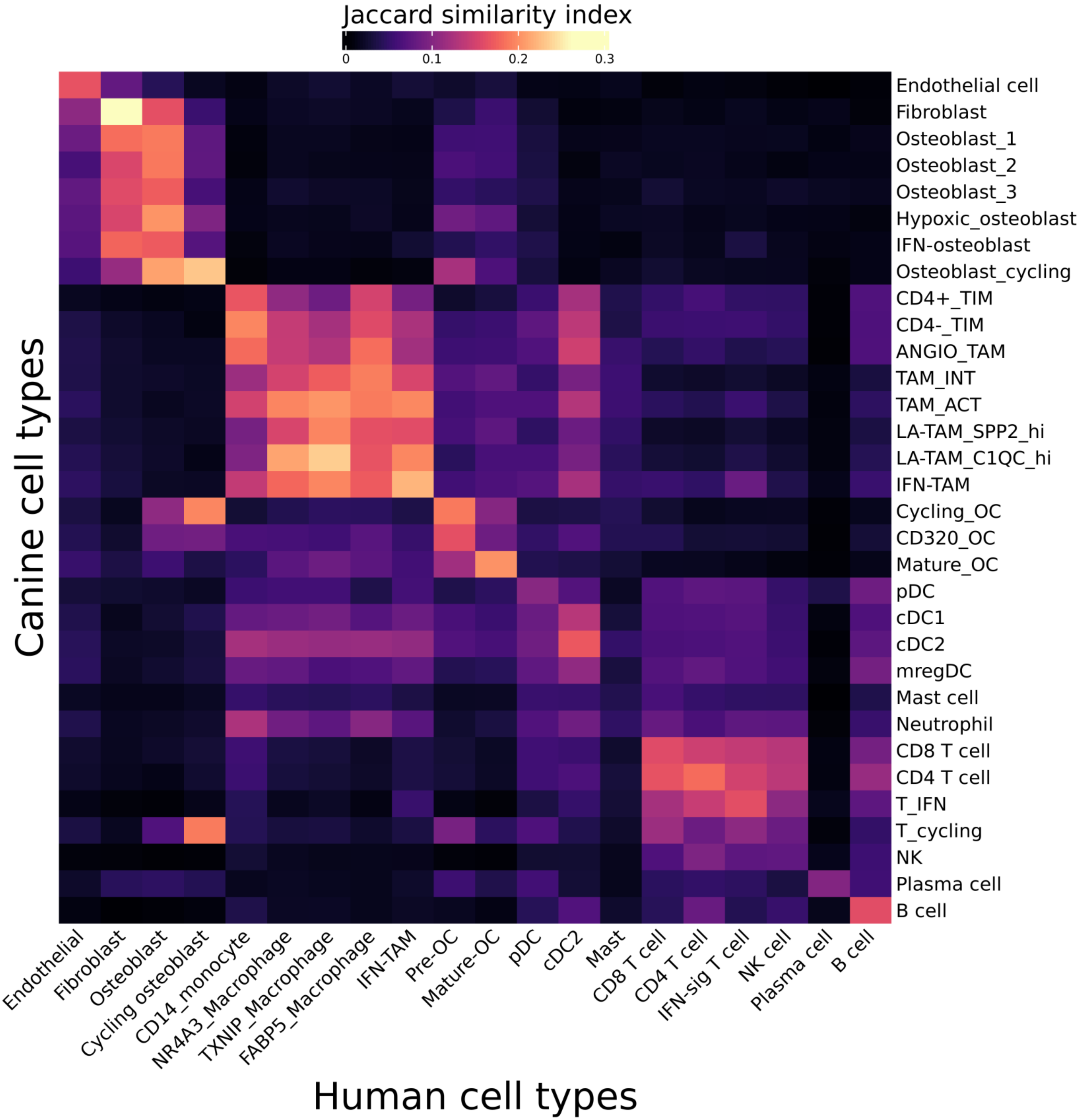

Supplemental figure 17. Heatmap depicting the Jaccard similarity index evaluating the degree of similarity between canine cell type gene signatures (rows) and human cell type gene signatures (columns).

Supplemental Table 1: Short gene lists generated curated from the results of FindAllMarkers().

**B cell**	
B cell	PAX5, CD22, MS4A1, FCRLA, CCR7, IGHM
Plasma cell	JCHAIN, DERL3, TNFRSF17, MZB1, POU2AF1
**T cell**	
CD4 naïve	CCR7, CD52, LTB, LEF1, TCF7
CD4 activated	CXCR4, CD28, IL2RB, IL7R, ICOS
CD4 follicular helper	TNFRSF18, TNFSF8, PDCD1, CXCL13, IL4I1
CD4 regulatory	IL2RB, GATA3, OCIAD2, ARID5B, IL21R
T-IFN	GZMA, ISG20, CCL5, IFI44L, OAS1
CD8 SPP1+	DNAJB1, HSP90AA1, FOS, HSPB1, C6H7orf50
CD8 effector	GZMB, NCR3, GZMA, CD96, FASLG, IL12RB2
CD8 exhausted	SEC14L1, PDCD1, GZMK, CCL5, CCL4
Cycling T cell	H1-5, MKI67, CENPF, SMC2, H2AZ1
**Dendritic cell**	
plasmacytoid DC	FCRLA, SPATS2L, IGKC, CLEC2D, RYR1, IGF1
precursor DC	FCRLA, PGLYRP2, DDR2, GPHA2, TCF4
mature regulatory DC	CCR7, FSCN1, IL1I1, MARCKSL1, CCL19, CD274
conventional DC subtype 1	CPNE3, CLEC1B, BATF3, SERPINB6, SMYD3
conventional DC subtype 2	CD300H, CD1C, PID1, LGALS3, MAFB
**Tumor**	
Hypoxic osteoblast	ENO1, PGF, PTGES, SFRP2, CAV1
Malignant osteoblast subtype 1	MPP6, LIFR, FBLN, NPY, C1S
Malignant osteoblast subtype 2	IBSP, SPARC, SMPD3, ALPL, WFDC1
Malignant osteoblast subtype 3	DNAJB1, HSP90AA1, FOS, HERPUD1, HSPH1
Cycling osteoblast subtype 1	UBE2S, DLGAP5, HMMR, TPX2, TUBA1B, CENPF
Cycling osteoblast subtype 2	TPX2, TK1, H1-4, RRM2, DNAJC9
Cycling osteoblast subtype 3	MCM6, RAD51AP1, HELLS, CDC6, UHRF1
Cycling osteoblast subtype 4	CDC20, PLK1, CENPE, MIK67, DLGAP5, NUF2
IFN-osteoblast	MX2, OAS1, IFI44, OAS2, IFI6
**Osteoclast**	
Mature osteoclast	CRYAB, ATP6V1C1, SLC4A2, CD84, NEURL3, HYAL1
CD320 osteoclast	VDR, HMGA1, APEX1, DDX21, RSL1D1
Cycling osteoclast 1/2	H2AZ1, STMN1, CENPF, CDC20, MKI67
**Monocyte**	
CD4- tumor infiltrating monocyte	CXCL8, VCAN, LYZ, PLBD1, LSP1
CD4+ tumor infiltrating monocyte	IL1B, PTGS2, LTF, THBS1, CXCL8, VCAN
**Macrophage**	
ANGIO-TAM	HBEGF, VEGFA, IL18BP, AREG, VEGFC
Intermediate TAM	CTSS, TPI, ENO1, LAMP2, CCL7
Activated TAM	CCL3, CD80, CCL19, CD5L, CXCL16, DLA-79
Lipid associated TAM (C1QC high)	C1QB, C1QC, PLTP, SERPING1, DAB2, CLDN1
Lipid associated TAM (SPP2 high)	TREM2, APOE, CD36, GPNMB, PRDX1
IFN-TAM	MX2, RSAD2, CCL8, CD40, IL7R, TNFSF10
**Miscellaneous**	
Neutrophil	SELL, SOD2, CXCL8, CD4, S100A8, PADI3
Mast cell	MS4A2, IL3RA, ADORA3, CSF2RB, ACE2, HPGD, CPA3
Fibroblast	DCN, IGFBP7, COL3A1, COL6A3, COL12A1, COL6A1
Endothelial cell	CD34, PLVAP, ESM1, EGFL7, FLT1, VWF

Supplemental Table 2: Breakdown of CellChat networks into “Immune”, “immune-related”, and “Non-immune” categories.

Immune	SPP1	CD45	CCL	SELPLG	CD86	SELL	IL1	CD80	PARs	IL16
	FASLG	FLT3	PD-L1	CD96	CD23	OSM	CD22	CADM	MPZ	BAFF
	THY1	ADGRE5	SEMA4	CD40	CSF					

Immune-related	GALECTIN	FN1	THBS	LAMININ	SEMA7	CLEC	BAG	RESISTIN	VISFATIN	NECTIN
	ICAM	NRG	NOTCH	HSPG						

Non-immune	COLLAGEN	APP	PTN	VEGF	BSP	PECAM1	IGF	FGF	CDH	TENASCIN
	APRIL	JAM	CDH1	ESAM	VWF	PERIOSTIN	CDH5	PDGF	NCAM	CALCR
	ANGPT	GAS								

Supplemental Table 3: QC metrics obtained from the Cell Ranger for each sample.

Sample	Dog ID	Estimated Number of Cells	Mean Reads per Cell	Median Genes per Cell	Number of Reads
tumor_no_tx_1_1	N1_1	6,747	83,085	1,996	560,571,180
tumor_no_tx_1_2	N1_2	7,266	85,063	2,002	618,070,887
tumor_no_tx_2_1	N2_1	9,163	56,345	855	516,292,820
tumor_no_tx_2_2	N2_2	9,806	61,951	898	607,489,392
tumor_no_tx_4	N3	5,897	84,552	2,052	498,602,137
tumor_no_tx_5	N4	6,570	75,654	1,358	497,048,147
no_tx_tumor_6	N5	7,389	73,085	1,366	540,021,401
tumor_no_tx_6	N6	5,181	88,923	780	460,707,555

Sample	Valid Barcodes	Sequencing Saturation	Q30 Bases in Barcode	Q30 Bases in RNA Read	Q30 Bases in UMI
tumor_no_tx_1_1	97.70%	55.80%	96.00%	92.10%	95.70%
tumor_no_tx_1_2	97.10%	59.40%	96.10%	91.90%	95.80%
tumor_no_tx_2_1	97.70%	56.90%	95.80%	91.30%	95.20%
tumor_no_tx_2_2	97.50%	56.00%	96.60%	93.00%	96.10%
tumor_no_tx_4	98.00%	59.00%	96.30%	92.70%	95.80%
tumor_no_tx_5	97.60%	57.00%	96.00%	91.20%	95.50%
no_tx_tumor_6	98.20%	55.00%	95.80%	90.90%	95.30%
tumor_no_tx_6	97.30%	74.70%	96.60%	91.50%	96.20%

Sample	Reads Mapped to Genome	Reads Mapped Confidently to Genome	Reads Mapped Confidently to Intergenic Regions	Reads Mapped Confidently to Intronic Regions	Reads Mapped Confidently to Exonic Regions
tumor_no_tx_1_1	93.30%	85.20%	19.70%	22.40%	43.20%
tumor_no_tx_1_2	92.60%	84.60%	18.80%	21.70%	44.00%
tumor_no_tx_2_1	94.50%	82.90%	19.30%	18.40%	45.20%
tumor_no_tx_2_2	94.70%	81.90%	19.50%	19.00%	43.50%
tumor_no_tx_4	95.10%	88.60%	17.80%	20.10%	50.80%
tumor_no_tx_5	94.00%	79.60%	18.70%	21.80%	39.10%
no_tx_tumor_6	93.30%	85.70%	18.50%	18.50%	48.70%
tumor_no_tx_6	94.80%	81.60%	20.40%	26.60%	34.60%

Sample	Reads Mapped Confidently to Transcriptome	Reads Mapped Antisense to Gene	Fraction Reads in Cells	Total Genes Detected	Median UMI Counts per Cell
tumor_no_tx_1_1	37.50%	0.70%	80.20%	16,474	7,934
tumor_no_tx_1_2	38.10%	0.80%	81.20%	16,514	7,945
tumor_no_tx_2_1	39.40%	0.70%	69.70%	15,942	2,405
tumor_no_tx_2_2	37.80%	0.80%	69.20%	16,139	2,452
tumor_no_tx_4	44.80%	0.70%	79.30%	16,304	8,790
tumor_no_tx_5	34.40%	0.50%	86.20%	16,222	5,287
no_tx_tumor_6	42.70%	0.50%	75.20%	16,515	5,274
tumor_no_tx_6	29.70%	0.60%	84.40%	15,297	2,347

Supplemental Table 4: Summary statistics (mean and range) of the data presented in Figure 3f.

Percent immune cells		
Cell type	Average	Range
CD4_naive	1.15	0.4-1.77
CD4_act	5.6	1.54-12.89
CD4_reg	4.89	1.63-10.55
CD4_fh	1.76	0.08-4.63
CD8_eff	4.89	2.46-9.8
CD8_ex	8.73	1.97-15.77
CD8_SPP1_hi	1.36	0.49-2.34
T_IFN	1.07	0.08-1.91
T_cycling	3.04	0.79-5.68
NK	0.28	0.06-0.9

Percent all cells		
Cell type	Average	Range
CD4_naive	0.77	0.28-1.65
CD4_act	3.74	0.58-7.89
CD4_reg	2.96	0.98-6.46
CD4_fh	1.12	0.05-2.83
CD8_eff	3.5	1.08-9.11
CD8_ex	5.65	1.18-9.66
CD8_SPP1_hi	0.81	0.45-1.43
T_IFN	0.68	0.05-1.35
T_cycling	1.89	0.47-3.41
NK	0.18	0.02-0.55

Supplemental Table 5: Gene signatures used to define "Pro-inflammatory" and "Anti-inflammatory" cell types in Figure 5e.

Pro-inflammatory	AZIN1	CD38	CD86	CXCL10	FPR2	GPR18	IL12B	IL18
	IRF5	NFKBIZ	NOS2	PTGS2	TLR4	TNF		

Anti-inflammatory	ALOX15	ARG1	CHIL3	CHIL4	EGR2	IL10	IRF4	KLF4
	MRC1	MYC	SOCS2	TGM2				

Supplemental Table 6: Summary statistics (mean and range) of the data presented in Figure 7c.

Percent immune cells		
Cell type	Average	Range
CD4-_TIM	4.36	0.91-7.23
CD4+_TIM	0.91	0.23-1.53
ANGIO_TAM	2.77	1.04-5.78
TAM_ACT	7.58	2.5-16.21
TAM_INT	5.54	0.65-10.88
LA-TAM_SPP2_hi	5.7	1.71-7.83
LA-TAM_C1QC_hi	4.81	1.47-6.41
IFN-TAM	1.43	0.43-3.22
CD320_OC	1.37	0.4-2.59
Cycling_OC	5.52	1.62-9.04
Mature_OC	2.87	0.4-9.45
cDC2	3.63	2.11-6.47
cDC1	0.98	0.17-3.08
mregDC	0.93	0.2-2.6
pDC	0.36	0.1-0.65
preDC	0.26	0.03-0.44

Percent all cells		
Cell type	Average	Range
CD4-_TIM	2.81	0.64-5.62
CD4+_TIM	0.56	0.17-0.94
ANGIO_TAM	1.6	0.83-3.47
TAM_ACT	4.7	1.95-11.48
TAM_INT	3.36	0.4-7.7
LA-TAM_SPP2_hi	3.37	1.59-4.94
LA-TAM_C1QC_hi	2.85	1.36-4.54
IFN-TAM	0.87	0.4-2.28
CD320_OC	0.79	0.24-1.56
Cycling_OC	3.2	1.5-5.43
Mature_OC	1.41	0.37-3.54
cDC2	2.16	1.46-3.88
cDC1	0.6	0.12-1.85
mregDC	0.6	0.14-1.56
pDC	0.23	0.06-0.43
preDC	0.16	0.02-0.31
