# Supplementary material for: Single-cell RNA sequencing reveals the cellular and molecular heterogeneity of treatment-naïve primary osteosarcoma in dogs
Source: Commun Biol. 2024 Apr 24;7:496. doi: 10.1038/s42003-024-06182-w (PMC11043452; doi:10.1038/s42003-024-06182-w)
Supplement: Supplementary file 3 — Description of Additional Supplementary Files [file 42003_2024_6182_MOESM3_ESM.pdf]

**Description of Additional Supplementary Files**

**File name:** Supplemental Data 1
**Description:** Results of FindAllMarkers() for all 41 cell types joined with the surfaceome database with “Surfaceome.Label” corresponding to the classification and “Surfaceome.Label.Source” indicating the method in which the prediction was made.

**File name:** Supplemental Data 2
**Description:** Short gene lists for all cell types described in the manuscript. Three different levels of annotation are provided (celltype.l1, celltype.l2, celltype.l3) and gene symbols are in column labeled “gene”. Same data as in Supplemental Table 1, but data are in long format.

**File name:** Supplemental Data 3
**Description:** Results of all pseudobulk contrasts presented in the manuscript in long data format. The contrast is indicated in the column “gs_base” and the “log2FoldChange” column direction is based on term1_VS_term2 as defined in the “gs_base” column.

**File name:** Supplemental Data 4
**Description:** Results of FindAllMarkers() on the T cell subsets joined with the surfaceome database with “Surfaceome.Label” corresponding to the classification and “Surfaceome.Label.Source” indicating the method in which the prediction was made.

**File name:** Supplemental Data 5
**Description:** Results of FindAllMarkers() on the myeloid subsets joined with the surfaceome database with “Surfaceome.Label” corresponding to the classification and “Surfaceome.Label.Source” indicating the method in which the prediction was made.

**File name:** Supplemental Data 6
**Description:** Source data used to generate the plots in Figure 9b.

**File name:** Supplemental Data 7
**Description:** Source data used to generate the plots in Figure 9c.

**File name:** Supplemental Data 8
**Description:** Source data used to generate the plots in Figure 9d.
