# Supplementary material for: Single-cell RNA sequencing reveals the cellular and molecular heterogeneity of treatment-naïve primary osteosarcoma in dogs
Source: Commun Biol. 2024 Apr 24;7:496. doi: 10.1038/s42003-024-06182-w (PMC11043452; doi:10.1038/s42003-024-06182-w)
Supplement: Supplementary file 12 — Reporting Summary [file 42003_2024_6182_MOESM12_ESM.pdf]

nature portfolio

Corresponding author(s): Dylan T Ammons
Steven Dow

Last updated by author(s): Jan 10, 2024

Reporting Summary

Nature Portfolio wishes to improve the reproducibility of the work that we publish. This form provides structure for consistency and transparency in reporting. For further information on Nature Portfolio policies, see our Editorial Policies and the Editorial Policy Checklist.

Statistics

For all statistical analyses, confirm that the following items are present in the figure legend, table legend, main text, or Methods section.

n/a	Confirmed	
☐	☒	The exact sample size (*n*) for each experimental group/condition, given as a discrete number and unit of measurement
☐	☒	A statement on whether measurements were taken from distinct samples or whether the same sample was measured repeatedly
☐	☒	The statistical test(s) used AND whether they are one- or two-sided<br>*Only common tests should be described solely by name; describe more complex techniques in the Methods section.*
☒	☐	A description of all covariates tested
☒	☐	A description of any assumptions or corrections, such as tests of normality and adjustment for multiple comparisons
☐	☒	A full description of the statistical parameters including central tendency (e.g. means) or other basic estimates (e.g. regression coefficient) AND variation (e.g. standard deviation) or associated estimates of uncertainty (e.g. confidence intervals)
☐	☒	For null hypothesis testing, the test statistic (e.g. *F*, *t*, *r*) with confidence intervals, effect sizes, degrees of freedom and *P* value noted<br>*Give P values as exact values whenever suitable.*
☒	☐	For Bayesian analysis, information on the choice of priors and Markov chain Monte Carlo settings
☒	☐	For hierarchical and complex designs, identification of the appropriate level for tests and full reporting of outcomes
☒	☐	Estimates of effect sizes (e.g. Cohen's *d*, Pearson's *r*), indicating how they were calculated

Our web collection on statistics for biologists contains articles on many of the points above.

Software and code

Policy information about availability of computer code

Data collection

Most analysis was completed in R software (v4.1.1; see session info below). Cell Ranger version 6.1.2 was used to aligned raw data to the CanFam3.1. The packages installed in the python environment used to run pySCENIC is provided after the R session info output.

```
R version 4.1.1 (2021-08-10)
Platform: x86_64-conda-linux-gnu (64-bit)
Running under: Red Hat Enterprise Linux 8.4 (Ootpa)

Matrix products: default
BLAS/LAPACK: /projects/dyammons@colostate.edu/software/anaconda/envs/r_env/lib/libopenblasp-r0.3.18.so

locale:
 [1] LC_CTYPE=C.UTF-8    LC_NUMERIC=C        LC_TIME=C
 [4] LC_COLLATE=C        LC_MONETARY=C       LC_MESSAGES=C
 [7] LC_PAPER=C          LC_NAME=C           LC_ADDRESS=C
[10] LC_TELEPHONE=C      LC_MEASUREMENT=C    LC_IDENTIFICATION=C

attached base packages:
[1] grid      stats4    stats     graphics  grDevices utils     datasets
[8] methods   base
```

```
other attached packages:
 [1] circlize_0.4.16           singleseqgset_0.1.2.9000
 [3] Matrix_1.5-1              copykat_1.0.5
 [5] infercnv_1.10.1           ComplexHeatmap_2.13.2
 [7] ggtree_3.2.1              ape_5.7-1
 [9] scuttle_1.4.0             scRNAseq_2.8.0
[11] ggpubr_0.4.0              slingshot_2.7.0
[13] TrajectoryUtils_1.2.0     SingleCellExperiment_1.16.0
[15] princurve_2.1.6           clusterProfiler_4.2.2
[17] msigdbr_7.5.1             ggsankey_0.0.99999
[19] lemon_0.4.5               reshape_0.8.9
[21] viridis_0.6.2             viridisLite_0.4.1
[23] SingleR_1.8.1             SeuratDisk_0.0.0.9019
[25] RColorBrewer_1.1-3        pheatmap_1.0.12
[27] DESeq2_1.34.0             SummarizedExperiment_1.24.0
[29] Biobase_2.54.0            MatrixGenerics_1.6.0
[31] matrixStats_1.0.0         GenomicRanges_1.46.1
[33] GenomeInfoDb_1.30.1       IRanges_2.28.0
[35] S4Vectors_0.32.4          BiocGenerics_0.40.0
[37] colorspace_2.0-3          ggrepel_0.9.1
[39] cowplot_1.1.1             scales_1.2.1
[41] patchwork_1.1.2           DoubletFinder_2.0.3
[43] clustree_0.4.4            ggraph_2.0.5
[45] forcats_0.5.2             stringr_1.4.1
[47] dplyr_1.0.10              purrr_0.3.5
[49] readr_2.1.2               tidyr_1.2.1
[51] tibble_3.1.8              ggplot2_3.3.6
[53] tidyverse_1.3.1           SeuratObject_4.1.3
[55] Seurat_4.3.0

loaded via a namespace (and not attached):
  [1] rsvd_1.0.5                  ica_1.0-3
  [3] Rsamtools_2.10.0            foreach_1.5.2
  [5] lmtest_0.9-40               crayon_1.5.2
  [7] MASS_7.3-58.1               nlme_3.1-160
  [9] backports_1.4.1             reprex_2.0.1
 [11] argparse_2.2.2              GOSemSim_2.20.0
 [13] rlang_1.0.6                 XVector_0.34.0
 [15] ROCR_1.0-11                 readxl_1.4.1
 [17] irlba_2.3.5                 limma_3.50.3
 [19] filelock_1.0.2              BiocParallel_1.28.3
 [21] rjson_0.2.21                bit64_4.0.5
 [23] glue_1.6.2                  sctransform_0.3.5
 [25] parallel_4.1.1              spatstat.sparse_3.0-0
 [27] AnnotationDbi_1.56.2        DOSE_3.20.1
 [29] spatstat.geom_3.0-5         haven_2.4.3
 [31] tidyselect_1.2.0            fitdistrplus_1.1-8
 [33] XML_3.99-0.12               zoo_1.8-9
 [35] GenomicAlignments_1.30.0    xtable_1.8-4
 [37] magrittr_2.0.3              phyclust_0.1-32
 [39] cli_3.6.0                   zlibbioc_1.40.0
 [41] rstudioapi_0.14             miniUI_0.1.1.1
 [43] sp_1.5-1                    rjags_4-13
 [45] fastmatch_1.1-3             lambda.r_1.2.4
 [47] ensembldb_2.18.3            treeio_1.18.1
 [49] shiny_1.7.1                 BiocSingular_1.10.0
 [51] xfun_0.39                   clue_0.3-62
 [53] cluster_2.1.4               caTools_1.18.2
 [55] tidygraph_1.2.2             KEGGREST_1.34.0
 [57] interactiveDisplayBase_1.32.0 listenv_0.8.0
 [59] Biostrings_2.62.0           png_0.1-7
 [61] future_1.29.0               withr_2.5.0
 [63] bitops_1.0-7                ggforce_0.4.1
 [65] plyr_1.8.7                  cellranger_1.1.0
 [67] AnnotationFilter_1.18.0     coda_0.19-4
 [69] pillar_1.8.1                gplots_3.1.3
 [71] GlobalOptions_0.1.2         cachem_1.0.6
 [73] GenomicFeatures_1.46.5      multcomp_1.4-20
 [75] fs_1.5.2                    hdf5r_1.3.8
 [77] GetoptLong_1.0.5            DelayedMatrixStats_1.16.0
 [79] vctrs_0.5.1                 ellipsis_0.3.2
 [81] generics_0.1.3              tools_4.1.1
 [83] munsell_0.5.0               tweenr_2.0.2
 [85] fgsea_1.20.0                DelayedArray_0.20.0
 [87] fastmap_1.1.0               compiler_4.1.1
```

```
 [89] abind_1.4-5                httpuv_1.6.5
 [91] rtracklayer_1.54.0         ExperimentHub_2.2.1
 [93] plotly_4.10.1              GenomeInfoDbData_1.2.7
 [95] gridExtra_2.3              edgeR_3.36.0
 [97] lattice_0.20-45            deldir_1.0-6
 [99] utf8_1.2.2                 later_1.3.0
[101] BiocFileCache_2.2.1        jsonlite_1.8.3
[103] ScaledMatrix_1.2.0         tidytree_0.3.9
[105] pbapply_1.5-0              carData_3.0-5
[107] sparseMatrixStats_1.6.0    genefilter_1.76.0
[109] lazyeval_0.2.2             promises_1.2.0.1
[111] car_3.1-0                  doParallel_1.0.17
[113] goftest_1.2-3              spatstat.utils_3.0-1
[115] reticulate_1.34.0          sandwich_3.0-2
[117] Rtsne_0.16                 downloader_0.4
[119] uwot_0.1.14                igraph_1.5.1
[121] survival_3.4-0             yaml_2.3.6
[123] htmltools_0.5.3            memoise_2.0.1
[125] modeltools_0.2-23          BiocIO_1.4.0
[127] locfit_1.5-9.6             graphlayouts_0.8.3
[129] digest_0.6.30              assertthat_0.2.1
[131] mime_0.12                  rappdirs_0.3.3
[133] futile.options_1.0.1       RSQLite_2.2.18
[135] yulab.utils_0.0.5          future.apply_1.10.0
[137] data.table_1.14.4          blob_1.2.3
[139] futile.logger_1.4.3        splines_4.1.1
[141] AnnotationHub_3.2.2        ProtGenerics_1.26.0
[143] RCurl_1.98-1.12            broom_1.0.1
[145] hms_1.1.2                  modelr_0.1.8
[147] BiocManager_1.30.19        shape_1.4.6
[149] libcoin_1.0-9              aplot_0.1.2
[151] coin_1.4-2                 Rcpp_1.0.11
[153] RANN_2.6.1                 mvtnorm_1.1-3
[155] enrichplot_1.14.2          fansi_1.0.3
[157] tzdb_0.3.0                 parallelly_1.32.1
[159] R6_2.5.1                   ggridges_0.5.4
[161] lifecycle_1.0.3            formatR_1.12
[163] curl_4.3.3                 ggsignif_0.6.4
[165] leiden_0.4.3               fastcluster_1.2.3
[167] DO.db_2.9                  qvalue_2.26.0
[169] TH.data_1.1-1              RcppAnnoy_0.0.20
[171] iterators_1.0.14           spatstat.explore_3.0-5
[173] htmlwidgets_1.5.4          beachmat_2.10.0
[175] polyclip_1.10-4            biomaRt_2.50.3
[177] shadowtext_0.1.1           timechange_0.1.1
[179] gridGraphics_0.5-1         rvest_1.0.3
[181] globals_0.16.1             spatstat.random_3.1-3
[183] progressr_0.11.0           codetools_0.2-18
[185] lubridate_1.9.0            GO.db_3.14.0
[187] gtools_3.9.3               prettyunits_1.1.1
[189] dbplyr_2.1.1               gtable_0.3.1
[191] DBI_1.1.3                  ggfun_0.0.8
[193] tensor_1.5                 httr_1.4.4
[195] KernSmooth_2.23-20         stringi_1.7.8
[197] progress_1.2.2             reshape2_1.4.4
[199] farver_2.1.1               annotate_1.72.0
[201] xml2_1.3.3                 BiocNeighbors_1.12.0
[203] restfulr_0.0.15            geneplotter_1.72.0
[205] ggplotify_0.1.0            scattermore_0.8
[207] BiocVersion_3.14.0         bit_4.0.4
[209] scatterpie_0.1.7           spatstat.data_3.0-0
[211] pkgconfig_2.0.3            babelgene_22.9
[213] rstatix_0.7.0              knitr_1.40

---

> pip list

Package                Version
---------------------- ---------
aiohttp                3.8.5
aiosignal              1.2.0
anndata                0.7.8
annoy                  1.17.3
arboreto               0.1.6
async-timeout          4.0.2
```

asynctest	0.13.0
attrs	22.2.0
backcall	0.2.0
bokeh	2.3.3
boltons	23.0.0
cached-property	1.5.2
certifi	2021.5.30
cffi	1.14.6
charset-normalizer	2.0.12
click	8.0.4
cloudpickle	2.2.1
contextvars	2.4
ctxcore	0.1.1
cycler	0.11.0
cytoolz	0.11.0
dask	2021.3.0
decorator	4.4.2
dill	0.3.4
distributed	2021.3.0
entrypoints	0.4
fa2	0.3.5
fbpca	1.0
frozendict	2.3.8
frozenlist	1.2.0
fsspec	2022.1.0
geosketch	1.2
get_version	2.1
h5py	3.1.0
HeapDict	1.0.1
idna	3.4
idna-ssl	1.1.0
immutables	0.19
importlib-metadata	4.8.3
importlib-resources	5.4.0
interlap	0.2.7
intervaltree	3.1.0
ipykernel	5.5.6
ipython	7.16.3
ipython-genutils	0.2.0
jedi	0.17.2
Jinja2	3.0.3
joblib	1.0.1
jupyter-client	7.1.2
jupyter-core	4.9.2
kiwisolver	1.3.1
legacy-api-wrap	1.2
llvmlite	0.36.0
locket	1.0.0
loompy	3.0.7
louvain	0.7.0
MarkupSafe	2.0.1
matplotlib	3.3.4
mkl-fft	1.1.0
mkl-random	1.1.0
mkl-service	2.3.0
mock	4.0.3
msgpack	1.0.5
MulticoreTSNE	0.1
multidict	5.2.0
multiprocessing-on-dill	3.5.0a4
natsort	8.2.0
nest-asyncio	1.5.7
networkx	2.5.1
numba	0.53.1
numexpr	2.7.3
numpy	1.19.5
numpy-groupies	0.9.22
olefile	0.46
packaging	21.3
pandas	1.1.5
parso	0.7.1
partd	1.2.0
patsy	0.5.1
pexpect	4.8.0
pickleshare	0.7.5
Pillow	8.3.1

pip	21.2.2
prompt-toolkit	3.0.36
psutil	5.9.5
ptyprocess	0.7.0
pyarrow	0.16.0
pycparser	2.21
Pygments	2.14.0
pynndescent	0.5.10
pyparsing	3.0.4
pyscenic	0.11.2
python-dateutil	2.8.2
python-igraph	0.9.6
pytz	2021.3
PyYAML	6.0.1
pyzmq	25.1.1
requests	2.27.1
scanorama	1.7.4
scanpy	1.7.2
scikit-learn	0.24.2
scikit-misc	0.1.4
scipy	1.5.3
seaborn	0.11.2
setuptools	58.0.4
sinfo	0.3.4
sip	4.19.25
six	1.16.0
sortedcontainers	2.4.0
statsmodels	0.12.2
stdlib-list	0.8.0
tables	3.6.1
tblib	1.7.0
texttable	1.6.4
threadpoolctl	2.2.0
toolz	0.11.2
tornado	6.1
tqdm	4.64.1
traitlets	4.3.3
typing_extensions	4.1.1
umap-learn	0.5.3
urllib3	1.26.16
wcwidth	0.2.6
wheel	0.37.1
xlrd	1.2.0
yarl	1.7.2
zict	2.1.0
zipp	3.6.0

Data analysis

All analysis code used to processed the data and generate figures can be found at: https://github.com/dyammons/canine_osteosarcoma_atlas and on Zenodo at https://zenodo.org/doi/10.5281/zenodo.10666968

For manuscripts utilizing custom algorithms or software that are central to the research but not yet described in published literature, software must be made available to editors and reviewers. We strongly encourage code deposition in a community repository (e.g. GitHub). See the Nature Portfolio guidelines for submitting code & software for further information.

Data

Policy information about availability of data

All manuscripts must include a data availability statement. This statement should provide the following information, where applicable:

- Accession codes, unique identifiers, or web links for publicly available datasets
- A description of any restrictions on data availability
- For clinical datasets or third party data, please ensure that the statement adheres to our policy

Raw sequencing data are available on the NCBI Gene Expression Omnibus database under the accession number GSE252470. The annotated dataset is available for browsing at the UCSC Cell Browser (https://cells.ucsc.edu/?ds=canine-os-atlas).

A project specific GitHub page containing all analysis code and software versions used to analyze the data presented in this manuscript is available at https://github.com/dyammons/canine_osteosarcoma_atlas. The analysis code and processed data (Seurat objects) are available on Zenodo (DOI: 10.5281/zenodo.10666968). Any additional data requests can be made by contacting a corresponding author.

Human research participants

Policy information about studies involving human research participants and Sex and Gender in Research.

Reporting on sex and gender	n/a
Population characteristics	n/a
Recruitment	n/a
Ethics oversight	n/a

Note that full information on the approval of the study protocol must also be provided in the manuscript.

Field-specific reporting

Please select the one below that is the best fit for your research. If you are not sure, read the appropriate sections before making your selection.

☒ Life sciences ☐ Behavioural & social sciences ☐ Ecological, evolutionary & environmental sciences

For a reference copy of the document with all sections, see nature.com/documents/nr-reporting-summary-flat.pdf

Life sciences study design

All studies must disclose on these points even when the disclosure is negative.

Sample size	No sample size calculation was completed prior to initiation of the study. Based on previous reports describing heterogeneity in human osteosarcoma (PMID: 34367994), we determined that 6 biological replicates of canine tumor samples would be adequate to capture tissue heterogeneity.
Data exclusions	All data collected in this study were used in analysis -- no data was excluded.
Replication	Biological replicates were used to complete the study, no experimental replication was used in this study.
Randomization	n/a
Blinding	The same pathologist completing histotyping of all tumor samples included in the study and they were blinded to any previous reports describing the tumor histotypes.

Reporting for specific materials, systems and methods

We require information from authors about some types of materials, experimental systems and methods used in many studies. Here, indicate whether each material, system or method listed is relevant to your study. If you are not sure if a list item applies to your research, read the appropriate section before selecting a response.

Materials & experimental systems

n/a	Involved in the study	
☒	☐	Antibodies
☒	☐	Eukaryotic cell lines
☒	☐	Palaeontology and archaeology
☐	☒	Animals and other organisms
☒	☐	Clinical data
☒	☐	Dual use research of concern

Methods

n/a	Involved in the study	
☒	☐	ChIP-seq
☒	☐	Flow cytometry
☒	☐	MRI-based neuroimaging

Animals and other research organisms

Policy information about studies involving animals; ARRIVE guidelines recommended for reporting animal research, and Sex and Gender in Research

Laboratory animals	n/a

Wild animals	n/a
Reporting on sex	Three male castrated (50%) and three female spayed (50%) dogs were used in the analysis presented in this manuscript.
Field-collected samples	Dogs for the study were selected based on the presence of an appendicular primary tumor and the absence of previous therapeutic intervention. All dogs presented with radiographic evidence of OS and subsequent histopathology was completed to confirm the diagnosis. Three male castrated and three female spayed dogs across a variety of breeds were included in the dataset. All study dogs underwent amputation of the affected limb and samples were collected for single-cell RNA sequencing processing within 30 minutes
Ethics oversight	All studies were approved by the Colorado State University (CSU) Institutional Animal Care and Use Committee and the CSU Clinical Review Board. We have complied with all relevant ethical regulations for animal use and all dog owners provided informed consent prior to sample collection.

Note that full information on the approval of the study protocol must also be provided in the manuscript.
